# A Lab‐On‐chip Tool for Rapid, Quantitative, and Stage‐selective Diagnosis of Malaria

**DOI:** 10.1002/advs.202004101

**Published:** 2021-05-13

**Authors:** Marco Giacometti, Francesca Milesi, Pietro Lorenzo Coppadoro, Alberto Rizzo, Federico Fagiani, Christian Rinaldi, Matteo Cantoni, Daniela Petti, Edoardo Albisetti, Marco Sampietro, Mariagrazia Ciardo, Giulia Siciliano, Pietro Alano, Brigitte Lemen, Joel Bombe, Marie Thérèse Nwaha Toukam, Paul Fernand Tina, Maria Rita Gismondo, Mario Corbellino, Romualdo Grande, Gianfranco Beniamino Fiore, Giorgio Ferrari, Spinello Antinori, Riccardo Bertacco

**Affiliations:** ^1^ Department of Electronics Information and Bioengineering Politecnico di Milano Piazza Leonardo da Vinci 32 Milano 20133 Italy; ^2^ Department of Physics Politecnico di Milano Piazza Leonardo da Vinci 32 Milano 20133 Italy; ^3^ Specialità di Microbiologia e Virologia Università degli Studi di Milano Milano Italy; ^4^ Dipartimento di Malattie Infettive Istituto Superiore di Sanità Viale Regina Elena n.299 Roma 00161 Italy; ^5^ Centre Médical Mgr Jean Zoa B.P. 185 Yaoundé Cameroon; ^6^ Hôpital Saint Luc B.P 50 Mbalmayo Cameroon; ^7^ UOC Microbiologia Clinica Virologia e Diagnostica Bioemergenza – Sacco teaching Hospital ASST FBF Sacco via GB Grassi Milano 74‐20157 Italy; ^8^ Department of Biomedical and Clinical Sciences “Luigi Sacco” University of Milano via GB Grassi Milano 74‐20157 Italy; ^9^ CNR‐IFN Institute for Photonics and Nanotechnologies Piazza Leonardo da Vinci 32 Milano 20133 Italy

**Keywords:** diagnostic tests, hemozoin nanocrystals, impedimetric detection, lab‐on‐chip, magnetic sorting, malaria, red blood cells

## Abstract

Malaria remains the most important mosquito‐borne infectious disease worldwide, with 229 million new cases and 409.000 deaths in 2019. The infection is caused by a protozoan parasite which attacks red blood cells by feeding on hemoglobin and transforming it into hemozoin. Despite the WHO recommendation of prompt malaria diagnosis, the quality of microscopy‐based diagnosis is frequently inadequate while rapid diagnostic tests based on antigens are not quantitative and still affected by non‐negligible false negative/positive results. PCR‐based methods are highly performant but still not widely used in endemic areas. Here, a diagnostic tool (TMek), based on the paramagnetic properties of hemozoin nanocrystals in infected red blood cells (i‐RBCs), is reported on. Exploiting the competition between gravity and magnetic forces, i‐RBCs in a whole blood specimen are sorted and electrically detected in a microchip. The amplitude and time evolution of the electrical signal allow for the quantification of i‐RBCs (in the range 10–10^5^ i‐RBC µL^−1^) and the distinction of the infection stage. A preliminary validation study on 75 patients with clinical suspect of malaria shows on‐field operability, without false negative and a few false positive results. These findings indicate the potential of TMek as a quantitative, stage‐selective, rapid test for malaria.

## Graphical abstract

Malaria is caused by the *Plasmodium* parasite which feeds on hemoglobin and produces paramagnetic hemozoin crystals within red blood cells (RBCs). A quantitative and stage‐selective pan‐malaria diagnostic test is disclosed, based on a simple concept implemented on a microchip: the sorting and electrical detection of infected RBCs, thanks to the competition between magnetic and gravity forces.

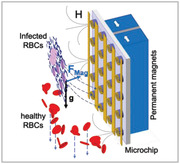

## 1 Introduction

Despite the strong efforts to eliminate malaria,^[^ ^1^, ^2^, ^3^ ^]^ this infection still represents a major cause of morbidity and mortality worldwide, with the highest burden of deaths recorded among children in sub‐Saharan Africa.^[^ ^4^ ^]^ Nowadays it is clear that to achieve the challenging objective of malaria eradication, an integrated strategy is required, involving developments in all relevant fields: vaccines,^[^ ^5^, ^6^ ^]^ antimalarial drugs, malaria transmission blocking, and diagnostics.^[^ ^7^ ^]^ Regarding diagnostics, even though the World Health Organization (WHO) strongly recommends prompt diagnosis before treatment, effective point‐of‐care diagnosis of malaria is still a challenge. Microscopy analysis, the time‐honored gold standard in malaria diagnostics, is incompatible with a wide screening of population in endemic zones due to the long operation times, operator dependent results, need for a performing optical microscope, and well trained microscopist.^[^ ^8^ ^]^ RDTs based on the antigens detection represented a real breakthrough, as they reduce the 40 min operating time of microscopy to about 15 min and do not require a qualified operator, but they still have a relatively high limit of detection (LoD) of about 200 parasites per µL (to be compared to about 20 parasites per µL for microscopy), are purely qualitative and do not distinguish between past and ongoing infections.^[^ ^9^, ^10^ ^]^ Furthermore, suboptimal performance of RDTs for detecting non‐falciparum species together with pfHRP2/3 gene deletions and delayed clearance dynamics for *Plasmodium falciparum* are responsible for a sizable number of false positive and negative results, particularly for non‐falciparum species and low parasitemia levels.^[^ ^11^, ^12^, ^13^, ^14^, ^15^ ^]^ Strategies to overcome gene deletion have been proposed, but quantification still remains a challenge. Emerging molecular tests based on DNA loop mediated isothermal amplification (LAMP) or similar technologies suitable for on‐field use are highly‐sensitive (LoD = 2 parasites per µL) but still not quantitative and rapid, as they require an operation time of about 60 minutes.^[^ ^16^, ^17^, ^18^ ^]^ For a recent review on advantages, limitations, and challenges of molecular methods toward a widespread usage in endemic areas, see ref. [19].

**Figure 1:**
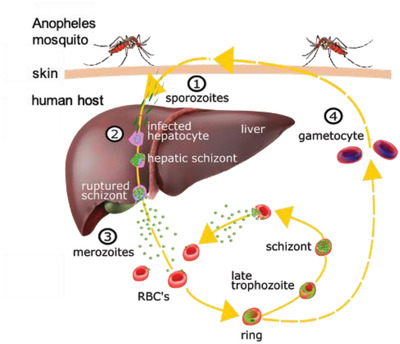
Malaria life cycle in the human body. Upon the Anopheles mosquito bite (1), sporozoites travel to the liver (2) where they infect hepatocytes and produce merozoites, till to the rupture of hepatic schizonts. Merozoites are thus delivered in the blood vessels (3) and infect red blood cells (RBCs), where the asexual reproduction takes place by subsequent stages (ring, trophozoite, schizont) till to the rupture of the cell membrane and delivery of new merozoites that infect other RBCs. Some of early stage trophozoites mature into gametocytes (4), the asexual form of *Plasmodium* which are taken up by the mosquito and transmit the infection, initiating the sexual reproduction within the Anopheles body.

Other emerging tests are based on the peculiar magnetic properties of corpuscles present in a blood sample from a patient affected by malaria. In the life cycle of *Plasmodium* (see **Figure** **1**), the motile form of the parasite (sporozoite) is injected in the human body during the bite of a female Anopheles mosquito and travels to the liver, where it reproduces asexually giving rise to thousands of merozoites which infect circulating RBCs. The parasite starts a series of asexual multiplication cycles (blood stage), with an RBC evolution by characteristic stages (ring, trophozoite, schizont), till rupture of the cell membrane and delivery of new merozoites which infect other RBCs. Some merozoites develop into immature gametocytes which are taken up by a mosquito during the hematic meal, mature within the mosquito gut, and give rise to female and male gametes. Gamete fusion leads to the formation of a motile ookinete, which traverses the insect midgut wall. In the hemocele, this transforms into an oocyst, in which thousands of sporozoites are formed and migrate to the salivary glands, where they are ready to be injected in the skin of the new individual at the subsequent bloodmeal, closing the parasite life cycle.

During the intra‐erythrocytic asexual stage of *Plasmodium* replication, the parasite feeds on hemoglobin and degrades it into free heme. This molecule is converted in an insoluble form, leading to the formation of nanometric hemozoin crystals (HC) with intrinsic volume magnetic susceptibility of +4 × 10^−4^.^[^ ^20^, ^21^, ^22^, ^23^, ^24^ ^]^ Noteworthy hemozoin is a very powerful malaria biomarker found in all erythrocyte stages of all clinically relevant *Plasmodium* species,^[^ ^17^, ^20^ ^]^ and various detection methods have been proposed.^[^ ^25^, ^26^, ^27^, ^28^, ^29^, ^30^, ^31^, ^32^, ^33^ ^]^ However, so far we did not assist to a widespread usage of hemozoin‐based tests. Most of in vitro assays require the RBC lysis to deliver free hemozoin crystals in the sample prior to quantification. Since the HC concentration can differ by more than two orders of magnitude in the various forms of i‐RBCs (see **Table** **1** showing the effect of such differences on the magnetic susceptibility), these methods may suffer from a large uncertainty in the quantification of the parasitemia (percentage of i‐RBCs) and the impossibility of identifying the parasite stages. In fact, the same amount of hemozoin could arise from a few gametocytes rich of hemozoin as well as from many rings poor of hemozoin which are typically found, respectively, at the end and at the beginning of the infection. Overall, a rapid diagnostic test suitable for on‐field quantification of low‐density parasites in population screenings, capable of distinguishing the asexual (rings) and sexual stages (gametocytes) found in peripheral blood, is still missing. Nevertheless, it would represent an invaluable tool to inform correct and personalized treatment of clinical cases and to identify healthy carriers in screening asymptomatic individuals, in view of human reservoirs elimination.

**Table 1:** Blood corpuscle magnetic volume susceptibility with respect to plasma

Corpuscle	Δ*χ* (x10^−6^)
Healthy oxygenated RBC^[^ ^52^, ^53^ ^]^	−0.18
Healthy RBC^[^ ^54^ ^]^	0.01
Infected RBC (ring stage)^[^ ^50^, ^55^ ^]^	0.82
Infected RBC (late trophozoite stage)^[^ ^50^, ^53^ ^]^	0.91
Infected RBC (schizont stage)^[^ ^50^, ^52^, ^53^, ^56^ ^]^	1.80
Fully deoxygenated RBC^[^ ^57^, ^58^ ^]^	3.33
MetHb‐RBC (t‐RBC)^[^ ^52^ ^]^	3.90
Infected RBC (gametocyte stage)	200
Hemozoin crystals (HC)^[^ ^18^ ^]^	410

Reported values are obtained from the references indicated. For gametocytes, the susceptibility has been estimated starting from that of HC, taking into account that in a gametocyte about half of its volume is filled by HC.^[^ ^59^ ^]^

Here, we present the first validation on human blood samples of a lab‐on‐chip diagnostic test exploiting the magnetic properties of hemozoin nanocrystals (the malaria pigment) based on a technology platform we recently introduced.^[^ ^34^ ^]^ On blood samples with calibrated concentrations of a synthetic model of i‐RBCs, giving almost the same signal of RBC infected by *P. falciparum* parasites in the late trophozoite stage, the test proved to be able to provide a direct and quantitative assessment of parasitemia in 10 min, with a detection sensitivity down to 10 i‐RBCs per µL and dynamic range up to 10^5^ i‐RBC per µL. Its name, TMek, comes from “Tid Mekii”, the name of malaria in the local language of Mbalmayo, the small village of Cameroon where a preclinical validation has been carried out. At variance with other tests based on hemozoin as biomarker, we developed a lab‐on‐chip physical method for the direct quantification of the level of parasitemia exploiting the magnetophoretic capture and impedimetric detection of intact i‐RBCs in a whole blood sample. As the impedance change produced by an i‐RBC captured between two electrodes is independent on its hemozoin content, the amplitude of TMek signal allows for a direct quantitative assessment of the parasitemia. In addition, the signal waveform is different for i‐RBCs at various stages and can be used to identify the parasite stage of development. In this paper, we show the test capability to distinguish among three stages (ring, late trophozoite, and gametocyte), even though for clinical applications most relevant ones are the ring pathogenic asexual stage and sexual mosquito transmissible stages (gametocytes). In fact, the peripheral blood of patients mainly contains rings and gametocytes, due to cytoadherence to the microvasculature and sequestration of i‐RBCs with mature developmental stages of *P. falciparum* (late trophozoite, schizont) within the organs of the human host. In this respect, the demonstration that TMek is capable of detecting and distinguishing rings, despite a smaller amount of hemozoin with respect to mature stages, is crucial to ensure the possibility of early stage detection. Finally, note that, even though our prevalidation has been mainly carried out on patients affected by *P. falciparum*, the transformation of hemozoin into hemoglobin is common to all *Plasmodium* species and the possibility of using TMek as a pan‐malaria test is an intriguing perspective to be investigated in the near future. The encouraging results reported on a well‐documented case of *P. vivax* from Sacco Hospital, as well as on one case of *P. malariae* and one case of *P. ovale* from Cameroon samples, confirm this perspective. Our method can be viewed as a point‐of‐care automatized version of microscopy, as it allows to directly count i‐RBCs and investigate the parasite stage. Especially in low‐resource settings, this could be of great help in the initial fast screening of patients under suspect of malaria or in the detection of healthy carriers, leading to prompt and personalized management of clinical cases while preventing asymptomatic individuals contributing to residual transmission.

## 2 Results

### 2.1 Assay Concept

**Figure 2:**
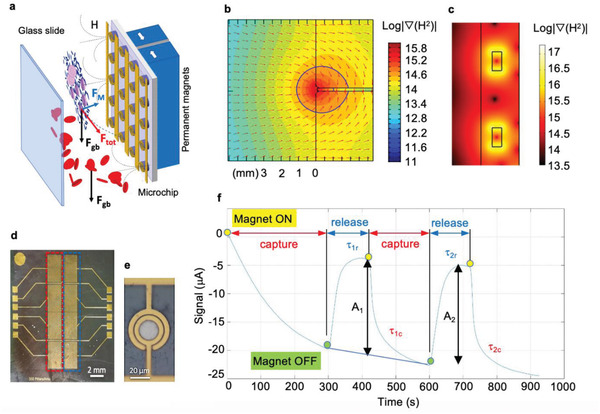
TMek concept and operating mode. a) Sketch of TMek concept. i‐RBCs (pink) and HC (black) are captured on cylindrical Ni concentrators, while healthy RBCs (red) sediment. b) Color plot of the macroscopic ∇*H* ^2^generated by the NdFeB permanent magnets with two north poles facing each other, and sandwiching a µ‐metal foil, as a function of the distance from their surface in a section plane perpendicular to the foil. Arrows indicate the magnetic force on paramagnetic corpuscles. c) Color plot of the localized ∇*H* ^2^close to the cylindrical Ni concentrators (black rectangles), in a plane perpendicular to the chip. d) Picture of the microchip with measurement (red) and reference (blue) areas. e) Top view of the annular gold electrodes on top of magnetic concentrators. Adapted with permission.^[^ ^33^ ^]^ f) Differential current signal versus time at fixed applied voltage amplitude, according to our measurement protocol: i) approach of external magnets (Magnet ON) and corpuscles capture during 5 min, ii) disengagement of magnets (Magnet OFF) and corpuscles release (2 min), iii) second approach of magnets and capture (3 min), iv) disengagement of magnets and release (2 min), v) third approach of magnets and capture (3 min).

Differently from other devices based on magnetophoretic separation of cells, like MACS columns and microfluidics devices, ^[^ ^35^, ^36^, ^37^, ^38^, ^39^ ^]^ we use micro magnetic concentrators for capturing the i‐RBCs on localized sites where a highly sensitive electrical detection is performed, thus allowing a quantification of the level of parasitemia on a single microchip, without any active microfluidics. The magnetophoretic force is given by $F_{m}=\frac{1}{2}\;\mu_{0}V\Delta \chi \nabla H^{2}$ where *µ*_0_ is the vacuum permeability, V the corpuscle volume, Δ*χ* the relative volume susceptibility of the corpuscle with respect to the medium, and ∇*H* ^2^ the gradient of the square of the magnetic field. Noteworthy, the characteristic Δ*χ* values for the corpuscles of interest with respect to the blood plasma reported in Table 1 show a remarkable difference between healthy and infected red blood cells. Fully oxygenated and healthy RBCs have Δ*χ* close to zero while infected RBCs display positive values which increase accordingly to the parasite stage due to accumulation of HC. This allows to implement a magnetophoretic separation of infected RBCs simply by exploiting the competition between gravity and magnetic force, as illustrated in **Figure** **2**a.

A silicon microchip (Figure 2d), with 1400 cylindrical magnetic Ni concentrators (see Figure 2e and Experimental Section), constitutes one wall of a microfluidic chamber containing the blood sample, whose volume is delimited by a parallel glass cover and a surrounding polymeric gasket of thickness *δ* = 500 µm. In the vertical configuration of Figure 2a, when the platform detects both free hemozoin and i‐RBCs but is more sensitive to i‐RBCs,^[^ ^33^ ^]^ all blood corpuscles sediment with a motion parallel to the chip surface. When an external magnet assembly is placed in close proximity to the back of the chip, a macroscopic magnetic field gradient is generated by the external magnets (∇*H* ^2^ ≈7 × 10^14^ A^2^ m^−3^, see Figure 2b), thus causing the attraction toward the chip of corpuscles with positive Δ*χ* (i‐RBC and free HC) while those with negative Δ*χ* (oxygenated healthy RBC, white blood cells and other blood corpuscles) are repelled. The strong localized field gradient created by the magnetic concentrators (see Figure 2c) finally allows to capture i‐RBCs and HC on top of them.

In view of a point‐of‐care use in endemic zones, we chose a simple and robust detection method to be integrated in a microchip: differential impedimetric detection between annular electrodes on top of the magnetic concentrators (measurement electrodes, Figure 2e) and the same number of electrodes, without concentrators underneath, placed nearby (reference electrodes), directly exposed to blood. This scheme allows an efficient subtraction of common mode spurious fluctuations. As both i‐RBCs and hemozoin crystals behave as insulating corpuscles in the MHz range,^[^ ^22^ ^]^ that is, in the frequency window used to detect the medium conductivity changes, the capture is associated with an impedance signal increase whose amplitude can be correlated to the corpuscle concentration. The signal waveform given by the capture rate, instead, reflects the whole corpuscle magnetic susceptibility and represents the fingerprint which can be used for stage identification.

Figure 2f reports the characteristic tracking of the differential current I(t) measured at 1 MHz applying a voltage stimulus of 100 mV to both measurement and reference electrodes after load of a whole blood sample diluted with heparin and PBS in the chamber, according to our measurement protocol including capture and release steps (see Experimental Section for details). The signal variation (A_1_) due to initial capture upon approach of the external magnet is better highlighted by the magnet disengagement after 5 min, causing corpuscles detachment and related sharp current increase, clearly distinguishable from spurious signal drifts during the capture. Noteworthy, the signal variation A_1_, proportional to the target corpuscles concentration, is evaluated just after 5 min from the beginning of the test, while the evaluation of the waveform for infection stage identification requires to acquire the signal for 10 min from the beginning. During the first preclinical study, sometimes the capture and release process has been repeated twice to improve the test reliability, especially at low parasite concentrations where spurious fluctuations can perturb the measurements. However, in view of a robust and uniform test assessment, only the A_1_ amplitude has been considered for quantification issues and test classification (positive/negative).

### 2.2 Quantification Capabilities

**Figure 3:**
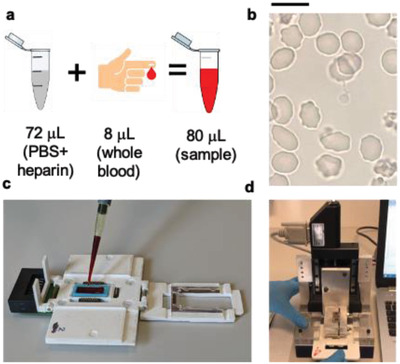
Sample preparation and load in the reader. a) Sample preparation consisting in whole blood dilution in a heparin‐phosphate buffered saline (PBS) solution (20 000 IU L^−1^). b) Optical microscope image of diluted blood, with hematocrit around 4%, showing the absence of RBCs aggregation. Scalebar: 10 µm. c) Load of the diluted blood sample on the glass slide within the cartridge holding the microchip in the lid. d) Upon closure of the lid, ensuring the cell sealing and the electrical connection via spring contacts, the cartridge is inserted in the reader.

The capabilities of TMek to quantify i‐RBCs and HC concentrations have been first evaluated using synthetic models with well‐known concentrations. For this purpose, RBCs from a healthy donor of Sacco Hospital have been treated with NaNO_2_ to induce a full transformation of hemoglobin into meta‐hemoglobin (see Experimental Section), thus obtaining treated RBCs (t‐RBCs from now on) mimicking the magnetic behavior of i‐RBCs (see Table 1),^[^ ^40^ ^]^ which were used for spiking a whole blood sample diluted (1:10) in PBS and heparin (see Experimental Section and **Figure** **3** for the sample preparation and test operation mode). Sample dilution helps magnetophoretic capture and reduces spurious signals from healthy blood corpuscles passing close to the electrodes during sedimentation.

**Figure 4:**
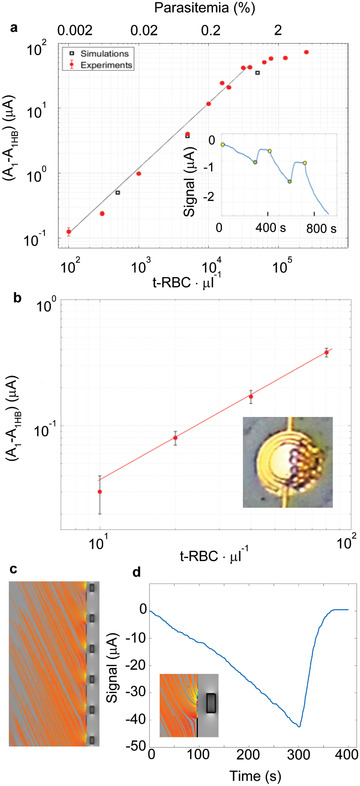
TMek performances on synthetic samples. a) Experimental calibration curve (red dots) on blood samples from healthy blood donors with known concentrations of t‐RBCs mimicking the behavior of mature RBCs infected by *Plasmodium*, compared to simulations (black squares). The experimental amplitude A_1_ measured after 5 min of capture, according to the protocol of Figure 2f, is plotted versus the t‐RBC concentration upon subtraction of the signal measured on diluted whole blood from a healthy donor without t‐RBCs (A_1WH_). Inset: typical waveform for t‐RBCs. b) Same as in (a), but for a capture time of 10 min, thus allowing to achieve a lowest detectable concentration of 10 t‐RBC per µL. Inset: optical image of captured t‐RBCs on top of the electrodes. Data in panels (a) and (b) are from one or two replicates for each concentration and error bars are derived from the signal SD, as explained in the methods. c) Trajectories of t‐RBCs under the action of the macroscopic and localized field gradients from COMSOL simulations. d) Simulated current signal versus time in the same experimental conditions of (a) for 1% parasitemia. Inset: zoom of the trajectories in proximity to a concentrator.

**Figure** **4**a shows the net signal, that is, the difference between the signal amplitude A_1_ due to t‐RBCs capture (see inset) and that measured from the diluted blood sample without t‐RBC (blank value on the order of 10–100 nA), as a function of the t‐RBC concentration. We observe a linear behavior over more than two decades (from 10^2^ to about 5 × 10^4^ t‐RBCs  per µL, corresponding to a parasitemia between 0.002% and 1%) followed by a change of slope at higher concentrations. The initial linear behavior, with a sensitivity S_5_ = 1 nA·(t‐RBC per µL)^−1^ fully reflects the expected proportionality between the resistance change (ΔR) and the volumetric fraction (*ϕ*) of insulating corpuscles in the proximity of the electrodes (see Note S3, Supporting Information). On the other hand, the saturation seen in Figure 4a can be easily explained by the filling of the probing volume at high parasitemia. Multiphysics simulation of the capture and detection process has been carried out with COMSOL (see Figure 4c,d and Experimental Section for details). The simulated amplitudes for different levels of parasitemia (0.01%, 0.1%, 1%) have been reported in Figure 4a with black squares, superposed to the experimental data. The agreement between experiments and simulations fully supports the consistency of our interpretation of the physical mechanism governing TMek test. Noteworthy, the detection sensitivity can be increased just by increasing the initial capture time. This is shown in Figure 4b, reporting a calibration curve where the amplitude A_1_ has been measured after 10 min from the beginning of the experiment. The sensitivity increases, up to the value S_10_ = 7 nA·(t‐RBCs per µL)^−1^, thus allowing to reach a lowest detectable concentration of 10 t‐RBCs per µL (10 parasites per µL) for 10 min capture time. These promising results indicate the possibility of reaching a limit of detection (LoD) on real samples from patients comparable with the best LoD achievable in microscopy and 20 times better than that of lateral flow RDTs.

**Figure 5:**
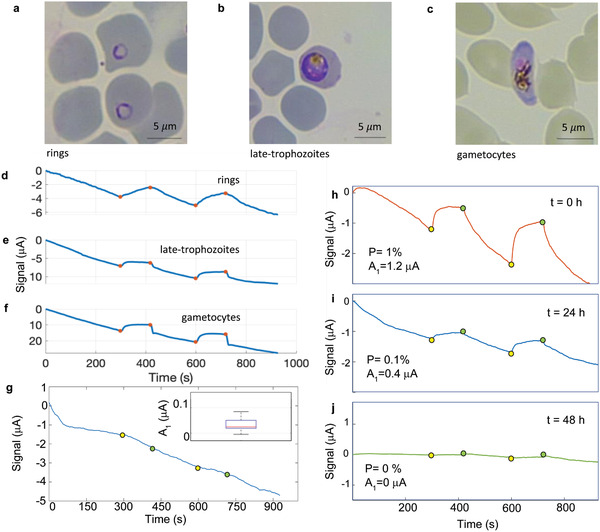
Experiments on infected blood samples. a–c) Optical microscopy images of stained i‐RBCs from in vitro cultivated *Plasmodium falciparum* parasites in the ring, late trophozoites and gametocyte stage. d–f) Waveforms measured on whole blood samples diluted (1:10) in parasite synthetic culture medium (RPMI 1640, pH 7.2) and heparin, with the same concentration of i‐RBC (about 5000 i‐RBC per µL) infected by *P. falciparum* at the stages corresponding to pictures in panels (a)–(c). g) Typical TMek signal measured on healthy blood donors and boxplot with the statistical analysis of A_1_ amplitudes from 10 healthy donors' blood samples (inset). The central mark indicates the median, and the bottom and top edges of the box indicate the 25th and 75th percentiles, respectively. h,i,j) TMek signal current versus time on blood sample from a patient affected by *Plasmodium vivax*, at *t* = 0 (admission at Sacco Hospital, parasitemia P = 1%), 24 h (P = 0.1%) and 48 h (negligible parasitemia) upon treatment with chloroquine.

The validity of our model based on t‐RBCs has been tested by performing some experiments on suspensions of i‐RBCs from in vitro cultivated *P. falciparum* parasites in the ring, late trophozoites, and gametocyte stage (see section 2.3 and **figure** **5**). From Figure 4a, it turns out that the signal for a concentration of 5000 t‐RBC µl^−1^ is 4 µA, to be compared with 4.5 µA and 4.4 µA measured on the same concentrations of cultivated rings and late trophozoites (see Figure 5d,e below). We observed only a slightly lower (10%) signal amplitude for t‐RBCs with respect to sexual forms that can be found in patient blood, thus confirming the validity of our model used in experiments for the determination of the calibration curve reported in Figure 4. In case of gametocytes with the same concentration (Figure 5f), the signal increases up to 6.8 µA, in agreement with their large magnetic susceptibility (see Table 1). Preliminary results on late trophozoites indicate a linear behavior with a detection sensitivity very similar to t‐RBCs, in agreement with the similarity of their volume magnetic susceptibility (see Table 1). The full calibration curve reported in Figure 4 can thus be used to estimate the sensitivity to late trophozoites, while further studies are needed to investigate the detection sensitivity to all stages of the parasite, in order to improve the quantification capabilities on real patient samples.

The sensitivity to free hemozoin has been evaluated using synthetic hemozoin crystals (*β*‐hematin) from Invivogen suspended in PBS (see Note S4, Supporting Information). We estimated a lowest detectable concentration of 3 µg mL^−1^, which corresponds to about 5000 parasites per µL,^[^ ^20^ ^]^ that is, two orders of magnitude larger than in case of direct detection of t‐RBCs. This is in good agreement with our initial design of electrodes (see methods), aiming to optimize the sensitivity to relatively large corpuscles like RBCs, instead of sub‐micrometric HC. Note also that free hemozoin is not commonly found in peripheral blood, as it mostly remains within the organs of the patient where the rupture of the infected RBC and the release of merozoites occurs.^[^ ^41^ ^]^ For this reason TMek has been explicitly designed to have a higher detectivity for integer i‐RBCs found in blood samples from patients.

### 2.3 Corpuscles Identification

To investigate the potential of TMek for stage‐identification we performed experiments on suspensions of i‐RBCs from in vitro cultivated *P. falciparum* parasites at different stages of development (rings at 10–16 h post RBC invasion, late trophozoites at 32–38 h post invasion and gametocytes at 10 days post invasion—see Experimental Section for details on the sample preparation and **Figure** **5**a–c for optical microscopy images of the various stages). Figure 5d–f reports TMek signals recorded for suspensions of rings, late trophozoites, and gametocytes at similar concentration, about 5000 i‐RBC per µL. Similar waveforms were found also at different levels of parasitemia, in the range 10^2^–10^5^ i‐RBC per µL.

The waveform from rings displays a smooth curve with slow signal variations upon magnet motion, while the signal rise and fall become steeper and steeper for trophozoites and gametocytes. The initial signal fall, due to capture from 0 to 300 s, is progressively more pronounced in rings, trophozoites and gametocytes, coherently with the increase of magnetic susceptibility of the i‐RBCs (see Table 1). The subsequent signal rise during the release does not depend on the corpuscle magnetic properties, as the magnetic field has been removed, strongly suggesting that the different dynamics measured between 300 and 420 s are associated to the different rheological properties of i‐RBCs at the various parasite stages,^[^ ^42^, ^43^ ^]^ reflecting their distinct morphologies (see Figure 5a–c). The impact of the corpuscles magnetic properties on the waveform is better seen during the second capture phase, after 420 s. In particular, looking at the signal fall at 420 s, for gametocytes we observe a sharp linear decrease ending with an undershoot before the signal level due to the drift is recovered. This is coherent with the fact that the magnetic susceptibility increases by two orders of magnitude from rings to gametocytes (see Table 1); the ratio between the magnetic and sedimentation force is much larger for gametocytes and, by consequence, the capture time after magnet re‐engagement is very short. As mentioned above, this reflects also in the dynamics from 0 to 300 s, but the first capture process is slow and waveform differences can be hindered by the signal drift. At 420 s, instead, magnetic corpuscles are still in the surroundings of the concentrators and the second capture is faster: the related signal can be easily disentangled from the drift and used as corpuscles fingerprint. While the signal waveform does not depend too much on the concentration, corpuscles identification becomes harder and harder when moving towards the limit of detection due to degradation of the signal to noise ratio, but our data show that this is possible at least down to about 1000 i‐RBC per µL. In conclusion, our results show that the waveform dynamics can be used to identify the stage of captured i‐RBC. The signal amplitude and the time evolution in response to the magnet disengagement/engagement provide complementary information related to the concentration of the i‐RBCs and to the development stage of the parasite, respectively. This is a peculiarity of TMek which makes it unique with respect to all other tests based on free hemozoin crystals as biomarker.

### 2.4 Tests on Human Blood Samples

The diagnostic platform has been first tested at the Sacco Hospital in Milano (Italy) and then in endemic zone during a first prevalidation carried out in April 2019 at the Hôpital Saint Luc of Mbalmayo (Cameroon). The sample preparation, simply consisting in the dilution of whole blood in PBS and heparin, and the test procedure are described in Figure 3 and in the methods.

We first tested TMek on 10 healthy blood donors at the Sacco Hospital (see Experimental Section for details). The typical signal waveform for a negative test is reported in Figure 5g, where no jumps are seen in correspondence of the magnet motion. The statistical analysis of the amplitude A_1_ is reported in the box‐plot of the inset in Figure 5g, showing a spurious average jump on the order of 30 nA, after the magnet disengagements (yellow dots) and engagements (green dots), mainly ascribed to the electromagnetic interference produced by the magnet approach/disengagement. Three cases of patients hospitalized for malaria have been studied in the laboratory of Sacco Hospital. In Figure 5h,i,j we report in particular the case of a patient affected by *Plasmodium vivax*, as confirmed by microscopy and PCR, during the illness evolution. TMek tests have been carried out on samples taken at the patient admission, 24 and 48 hours after the beginning of the treatment with chloroquine. Microscopy analysis indicates an initial parasitemia of 1%, which decreases to about 0.1% after 24 h and is negligible 48 h after beginning of the treatment. The corresponding TMek signals show an initial amplitude A_1_ of 1 µA, gradually decreasing to zero at 48 h, when a residual signal (A_2_) is seen only in the second release due to the longer capture time. This preliminary experiment indicates the potential of TMek as valuable fast and quantitative tool to be used not only for diagnostics but also for monitoring the illness evolution during treatment.

**Table** **2** reports the results of the preclinical study carried out in Cameroon on 75 patients mainly affected by *P. falciparum*, using a reference classification carried out by Infectious Diseases specialists on the basis of three independent light‐microscopy evaluations and additional clinical data of patients (see methods for details). Within the limits of this preliminary study with large 95% confidence intervals (in brackets below), the lack of false negative results for TMek indicates a high sensitivity, 100% (93.3–100.0), both for peripheral (venous) and capillary samples. This is crucial to avoid that a patient affected by malaria is not specifically treated with antimalarials. Nine false positive are found on a total number of 29 patients not affected by malaria in venous samples, leading to a specificity of 69% (49.2–84.7). On the contrary, we did not find false positive results in case of samples from a finger prick, at least within the limited number of these samples (just 10 due to time limitations in the first prevalidation campaign). The different results obtained for venous and capillary sampling are not surprising because venous samples were analyzed up to 16 h after sampling. In that period, a sizable degradation of hemoglobin can occur, with partial transformation into paramagnetic methemoglobin which produces a RBC magnetic susceptibility similar to that of i‐RBCs. Blood samples from a finger prick, instead, were analyzed within 5 h after sampling. Despite the limited number of finger prick samples (10) and related large confidence intervals in Table 2, our results indicate that TMek holds promise to display both a very high sensitivity and specificity in the form of a prick‐test, as expected for a point‐of‐care diagnostic tool.

**Table 2:** Comparative performances of TMek and RDT

Result	TMek (venous)	TMek (capillary)	RDT (SD Bioline)
True positive (n)	46	8	45
False positive (n)	9	0	5
False negative (n)	0	0	1
True negative (n)	20	2	24
Total number of tests	75	10	75
Sensitivity (% [95% CI])	100.0 (93.3–100.0)	100.0 (63.0–100.0)	97.8 (88.5–99.9)
Specificity (% [95% CI])	69.0 (49.2–84.7)	100.0 (15.8–100.0)	82.7 (64.2–94.1)
Negative predictive value (%)	100.0	100	96.0 (77.42–99.41)
Positive predictive value (%)	83.6 (74.8–89.8)	100	90.0 (80.2–95.2)

Results of TMek and of a lateral‐flow RDT (SD BIOLINE MALARIA Ag P.F/PAN, ABBOT) from the preclinical study in Cameroon. All positive patients were identified as affected by *P. falciparum* except one case of *P. malariae* and one case of *P. ovale*.

## 3 Discussion

The results reported in Section 2.2 show that TMek can provide a quantitative assessment of the parasitemia over more than three decades, spanning the entire range of interest for a fast diagnosis of malaria by clinicians. The lowest detectable concentration for a capture time of 5 min, according to Figure 4a, is in the order of 100 t‐RBCs per µL (corresponding to 100 equivalent parasites per µL, as a RBC is infected by a single parasite). The detection limit can be reduced down to 10 t‐RBCs per µL just by doubling the capture time and even higher sensitivity could be achieved by further increasing the test operating time. Playing with the capture time offers an easy way to tune the dynamic range and sensitivity for an accurate monitoring of the parasitemia during the clinical treatment of the disease or the identification of low‐level malaria parasites in asymptomatic people. Experiments on different stages of cultivated *Plasmodium falciparum* parasites, reported in Section 2.3, demonstrated instead the possibility of distinguishing i‐RBCs in the ring, late trophozoite, and gametocyte stage looking at the peculiar signal dynamics (waveform), which can be used as stage‐fingerprint. Although it is beyond the scope of the present work, we note that the combined waveform analysis in terms of both amplitude and dynamics could allow disentangling and quantifying i‐RBCs containing parasites at different stages of development in human blood samples, providing clinicians with a comprehensive assessment of the infection evolution. This could be crucial to monitor the effect of treatment in clinical case management and determine the parasite potential transmissibility to mosquitoes associated to gametocytes in population screening, particularly in the case of *P. falciparum* infections, where the only parasite stages in peripheral circulation are the clearly distinguishable rings and mature gametocytes. In this framework, the test could enable new strategies for personalized malaria treatment and community‐based screening aiming at the identification of asymptomatic malaria‐infected individuals (rings, trophozoites, gametocyte carriers) to be specifically treated to block the infection transmission.

The preliminary study in Cameroon reported in Section 2.4 confirms the on‐field operability of TMek. For a rough comparison of TMek with state‐of‐the‐art rapid diagnostic test, the fourth column of Table 2 reports the statistical analysis of results form a commercial antigen‐based RDT performed in parallel on the same patients samples used for TMek prevalidation. Both false positive and negative results are found, consistent with well‐known issues related to RDTs.^[^ ^44^ ^]^ Interestingly enough, the estimated values of sensitivity and specificity of this well‐established RDT are comparable to those of the very first prototype of TMek, within the limits of the large confidence intervals of this study, thus indicating the potential of our method. In order to validate these preliminary results, we are planning a new campaign with an increased number of samples comparing RDTs, Microscopy, and TMek using PCR as the reference method.

A first analysis of the on‐field quantification capability of TMek is reported in Figure S7 and Note S5, Supporting Information. Just a moderate linear correlation between the TMek signal amplitude and the parasitemia from microscopy evaluation is found, with a Pearson correlation coefficient about 0.5. The fact that the test is not fully quantitative on patient samples is partially due to the large error bars both on the parasitemia and the signal amplitude, the latter also associated to the use of different chips and setups in the on‐field tests. Note also that, in the present analysis, the signal amplitude was not normalized to the detection sensitivity of the specific parasite stage in the patient blood sample, as arising from full calibration curves on the different stages, yet not available. The results on the corpuscle identification reported in Section 2.3 suggest that, in future developments of the test, a deconvolution of the signal waveform into characteristic waveforms for the various parasite stages could be performed, possibly via application of patterning recognition routines. A more reliable quantification of the parasitemia could then be obtained by normalizing each characteristic waveform to the specific sensitivity. A careful engineering of the measurement apparatus, chip‐production scale‐up and an advanced data analysis are needed to improve the on‐field test quantification capability. However, the promising laboratory results obtained on synthetic samples of malaria‐infected blood (Figure 4) indicate the potential of TMek to become fully quantitative also in on‐field applications. The estimated test cost for a production of 10 millions disposable chips + cartridge/year is on the order of $1.5, with minimal impact of the reader cost (about 100 $) as it can be reused thousands of times. TMek cost is higher than that of lateral‐flow RDTs ($0.2–0.4 for large volumes in endemic areas)^[^ ^45^ ^]^ but definitely lower than that of molecular methods and also microscopy, when considering the labor time and training costs of expert microscopists. Even in comparison with RDTs, however, the difference of cost could be justified for specific target markets or screening campaigns requiring the intriguing features of our test combined in an engineered version of TMek suitable for application at the patient bedside.

Finally let us mention possible interferences on the test performances coming from other parasites producing hemozoin, like Schistosoma, and other pathologies favoring the transformation of hemoglobin into methemoglobin (sickle cell disease, methemoglobinemia). The investigation of TMek response to samples from patients with these pathologies is a crucial point of future studies in order to find related fingerprints and asses the test selectivity.

## 4 Conclusion

To summarize, we reported on a quantitative and stage‐selective rapid diagnostic test with great potential for low density parasite screening, therapy monitoring, and transmission blocking. Laboratory tests on synthetic models of malaria infected blood show that, after 5–10 min of capture time, TMek can provide a direct and quantitative evaluation of the parasitemia, in the range 10–10^5^ parasites per µL. A preclinical study carried out in Cameroon, on 75 patients, showed a sensitivity and specificity of 100% (93.3–100.0) and 69% (49.2–84.7) with respect to a reference classification from microscopy and clinical data, comparable to those of RDTs used in parallel, within the large uncertainty intervals associated to the limited number of samples of this proof‐of‐concept study. Preliminary results indicate that specificity could be increased by reducing the delay between the blood sampling and the analysis, in the typical conditions foreseen for a lab‐on‐chip finger‐prick test to be used on‐field. These findings, together with the detection capability of both *P. falciparum* and *P*. *vivax*, point to the potential of TMek to become the lab‐on‐chip equivalent of microscopy, providing clinicians with an easy, fast, automatic and operator‐independent tool for on‐field quantitative assessment of the parasitemia, of the disease progression and of the potential human‐to‐mosquito parasite transmissibility.

## 5 Experimental Section

### External Magnets and Ni Concentrators

Even though in the present work only results in the vertical configuration are presented, where the magnetic and gravity forces are perpendicular, the apparatus can be operated also in the horizontal configuration where the magnetic force opposes gravity. The right order of magnitude for the *∇H^2^* needed to efficiently capture i‐RBCs during their sedimentation can be easily estimated with reference to the horizontal configuration, by equating the magnetic force and the gravity‐buoyancy force. This leads to typical values of *∇H^2^* about 1 × 10^15^ A^2^ m^−3^ for i‐RBCs and 1.7 × 10^13^ A^2^ m^−3^ for HC (see Note S1, Supporting Information). To achieve these values, a magnet assembly made of two NdFeB (N52 grade) permanent magnets, in shape of parallelepipeds with 6 × 25 × 25 mm^3^ size, was realized, mechanically clamped to sandwich a *µ*‐metal foil (0.2 mm thick) with the faces having the same polarity. This configuration allowed to concentrate a strong magnetic field in the *µ*‐metal foil and produced a high gradient at its edges. Experimentally (see Note S1, Supporting Information), the *∇H^2^* at 0.5 mm from the magnet surface, that is, at the typical distance of the concentrators from the magnet when the latter is placed in close proximity to the back of the chip, was 7 × 10^14^ A^2^ m^−3^, that is, on the same order of magnitude of the threshold values listed above. Ferromagnetic concentrators made of Ni were designed in order to easily saturate in the field by the external magnets, while displaying a negligible remanence. Characteristic magnetic hysteresis loops are reported in Figure S1, Supporting Information. Capture simulations allowed to define a suitable geometry for the array of concentrators, maximizing the number of captured cells while keeping as low as possible their surface density on the chip. This allowed localizing the cells on a small area covered by the electrodes for the impedimetric detection increasing the effect of a single cell on the total electrical signal. The optimized array layout (see Figure S2, Supporting Information) was made of Ni cylinders (with 40 µm diameter and 20 µm height) arranged in a hexagonal lattice with lattice parameter of 160 µm. The local *∇H^2^* at the concentrator surface in saturation was on the order of 10^16^ A^2^ m^−3^, larger than the macroscopic one produced by external magnet, thus allowing for efficient concentration on top of measurement electrodes.

### Microchip Layout and Fabrication

The Si microchip of Figure 2d has an overall area of 18 × 22 mm^2^ and allowed to measure four equivalent sensors in parallel, to improve statistics. Each sensor corresponds to a couple of areas of 2 × 4 mm^2^ with measurement and reference electrodes aligned horizontally in Figure 2d. A couple of coplanar gold electrodes were fabricated on top of each concentrator for a total of 350 couples connected in parallel in each sensor area. An identical number of reference electrodes are placed nearby, in the corresponding reference area, without magnetic concentrators underneath. Since the i‐RBCs were attracted on the edges of the Ni cylinders where the magnetic gradient is maximum, the electrodes had an annular shape (Figure 2e) with width and spacing of 3 µm, suitable for the detection of RBCs with typical diameter of 8 µm. The geometry of the electrodes was optimized to minimize the sensitive area, that is, the area between the couples of electrodes, thus maximizing the impedance change given by a single cell (see Note S3, Supporting Information).

Two out‐of‐phase sinusoidal voltage signals of 100 mV at 1 MHz were applied to the outer elements of the reference and measurement annular electrodes, while the inner elements were all connected to the virtual ground of a transimpedance amplifier (see Figure S4, Supporting Information). In this way, the net differential current arising from the impedance variation upon corpuscle capture on the measurement electrodes was measured upon subtraction of spurious fluctuations from the reference.

The electrical connections of the electrodes to the board on the cartridge were realized via spring contacts matching the position of the outer pads in the chip. The chip fabrication was carried out using microfabrication techniques and materials compatible with standard silicon foundries to achieve a cost‐effective high‐volume manufacturing. Specifically, the chips were fabricated starting from heavily p‐doped 4” Si wafers (P+/Boron–0.005–0.025 Ohm cm) through the following steps: i) realization of the hexagonal array of 20 µm deep cylindrical cavities via reactive ion etching through a 20 µm thick positive photoresist with photolithographically defined circular holes; ii) filling by electroplating using a silicon wafer plating laboratory set (A‐52‐ST4W‐YTC300) provided by “Yamamoto MS” and Ni sulfamate solution (Spherolyte Ni TSV) provided by ATOTECH; iii) chip planarization via mechanical polishing; iv) deposition of a 3 µm thick SiO_2_ layer by plasma enhanced chemical vapor deposition (PECVD) to electrically isolate the magnetic and electric layers; v) fabrication of the gold electrodes (Au 200 nm/Cr 30 nm) by lift‐off; vi) selective passivation of the wiring from the pads to the electrodes with a 5 µm photoresist (SU8), to reduce the background signal not arising from the electrodes. Finally the chips are coated with a co‐polymer (dimethylacrylamide (DMA), N‐acryloyloxysuccinimide (NAS) and 3‐(trimethoxysilyl) propyl methacrylate (MAPS), copoly(DMA‐MAPS‐NAS))^[^ ^46^ ^]^ to improve the surface hydrophilicity. For further details see Supporting Information, Note 2.

### Electronic Platform for Impedimetric Detection and Data Acquisition

The impedimetric detection was performed by a custom compact circuit board carefully designed for a low noise operation and a low cost. Two counter‐phase voltage signals at 1 MHz were generated by a direct digital synthesizer (DDS) and applied to the measurement and reference electrodes of the sensor (Figure S4 and Note S3, Supporting Information). A transimpedance amplifier (TIA) was connected to the common node of the sensor in order to read the current and keep the electrode at 0 V. If the impedances of measurement and reference areas were equal, no current was measured by the TIA. The capture of i‐RBCs on the measurement electrodes caused an imbalance of the current which was amplified by the TIA. The amplitude of the sinusoidal voltage applied to the electrodes was limited to 100 mV in order to avoid electrochemical reaction at the metal–liquid interface. Before each experiment, an automatic tuning of the counter‐phase voltage applied to the reference electrode assured a well‐balanced starting condition. The electronic board implemented an analog lock‐in amplifier to extract the real part of the differential admittance of the sensor at 1 MHz. The resulting signal was low‐pass filtered at 100 Hz, sampled by a low power 16‐bit analog‐to‐digital converter, and further filtered in the digital domain by the on‐board microcontroller (Arduino Due). The common nodes of four sensors fabricated on the silicon chip were connected to the input TIA through a low‐on resistance multiplexer. The microcontroller sequentially scanned the four sensors with a frequency of 1 Hz. The full system was able to detect a variation of about 8 ppm of the nominal impedance of the single‐ended sensor (170 Ω at 1 MHz), well below the spurious impedance variation given by the movement of the permanent magnet and by the conductivity drift that currently limit the minimum detectable signal.

The data acquisition and control of the full experiment was managed through an USB connection by a custom software developed in Matlab using App Designer. It allowed to set the measurement parameters, start/interrupt the experiment, acquire the signal of the sensors, control the movement of the permanent magnet, and visualize in real time the test output.

### Preparation of t‐RBCs Mimicking the Behavior of Infected Red Blood Cells

Whole blood samples were centrifuged for 3 min at 3000 rpm at room temperature. Native plasma was collected and the remaining particulate was washed by a repeated resuspension–centrifugation sequence. After the third centrifugation, the buffy coat was removed using a 5‐mL Pasteur pipette. The buffy coat removal sequence was then repeated twice. The resulting RBCs layer was finally resuspended in PBS (Calcium&Magnesium free PBS, Euroclone Italia, Milan, Italy) to a hematocrit (Hct) of 40%, which was measured by capillary centrifugation at 12 000 rpm for 5 min. The RBCs suspension was then fully oxygenated by recirculating the sample in a 1 m long, 0.8 mm I.D., 0.8 mm wall thickness silicone tube for 30 min at 8 mL min^−1^ using a peristaltic pump, at room temperature. After oxygenation, the RBC suspension was washed again by repeated resuspension‐centrifugation in order to remove possible hemolysis products, due to pump mechanical stresses and RBC time‐related degradation.

Sodium nitrite (NaNO_2_) was used to produce methemoglobin in suspended RBCs.^[^ ^47^ ^]^ A 80%‐Hct RBC suspension was gently diluted with an equal volume of a pre‐prepared solution of NaNO_2_ in PBS (2 mg mL^−1^). The obtained suspension (Hct = 40%) was rocker‐incubated for 30 min at room temperature. After treatment, the solution was washed thrice with PBS to remove the remaining NaNO_2_ and brought to a final Hct of 4%.

Samples with well‐known simulated parasitemia used for the calibration curves reported in Figure 4 were obtained by spiking the whole blood by the same healthy donor, from which RBCs were extracted and treated, with t‐RBCs at various concentrations and then diluting with PBS and heparin to achieve a final hematocrit of about 4%.

### Simulations

Multiphysics simulations were carried out with COMSOL Multiphysics 5.3a in order to shed light on the mechanism of magnetophoretic capture. The simulation field was a portion of the chip (1400 × 400 µm) including 13 Ni concentrators, in contact with a 500 µm layer of RBCs suspended in PBS, like in real experiments where the gasket defining the lateral wall of the magnetophoretic cell is 500 µm high. The volume was uniformly filled above the chip with the proper number of t‐RBCs per µL corresponding to each parasitemia considered in this study. Each t‐RBCs was modeled as a spherical particle with volume *V_p_* = 5.8 × 10^−17^ m^3^, mass *m_p_* = 6.6 × 10^−19^ kg, magnetic volume susceptibility with respect to plasma Δ*χ* = 3.9 × 10^−6^. The approximation of RBCs to spherical particles to model their motion in viscous fluid is a common approach valid for non‐interacting cells,^[^ ^48^ ^]^ as in this experimental setting where the initial blood dilution was expected to reduce the interaction between blood particles in the suspending fluid to a negligible factor. The magnetic field gradient produced by the Ni concentrators was first simulated by COMSOL (Figure 2c) and then used in the particle tracing module to calculate the local attracting force toward the concentrators. The action of the macroscopic field gradient produced by external magnets was modeled by a uniform force within the simulation field, corresponding to the magnetophoretic force on a t‐RBCs due to the average *∇H^2^* value of 4 × 10^14^ A^2^ m^−3^ measured from 0.5 to 1 mm distance from the magnets surface, corresponding to the volume occupied by blood in the TMek apparatus during capture. The current variation upon t‐RBCs capture was evaluated by monitoring over time the number of t‐RBCs entering a plane placed at 20 µm from the chip surface, assuming that from that time they contribute to the volumetric fraction of insulating corpuscles in the probing volume of electrodes. As a matter of fact, their capture on the electrodes from that distance was very fast and this avoided computational issues related to the mesh thickening close to the concentrators. According to Maxwell formula (see Supporting Information), the impedance variation is proportional to the volume fraction of the corpuscles, so that the current variation during capture can be estimated with reference to the specific experimental conditions (sensor impedance of 170 Ω and applied voltage amplitude of 100 mV). To evaluate the current profile during the release induced by the magnet disengagement, from 300 to 420 s, it was started from the configuration of captured t‐RBC at 300 s previously obtained and simulated the detachment due to Brownian motion and gravity in the absence of magnetic field gradients. Note that in real blood samples, i‐RBCs in the ring stage tend to be biconcave, trophozoites are more globular while gametocytes have a characteristic banana‐shape. These differences can produce some modifications in the viscous force leading to a modified dynamics of the magnetophoretic capture. On the other hand, the amplitude of the impedimetric signal is not expected to change too much as, to the first order, it depends on the volume, not on the shape of the corpuscle.

### Sample Preparation of RBCs Infected In Vitro by Different Blood Stages of P. falciparum

*P. falciparum* parasite line NF54^[^ ^49^ ^]^ was cultivated by standard methods^[^ ^50^ ^]^ in O+ RBCs in RPMI 1640 medium (Gibco) supplemented with 25 mm Hepes, 50 µg mL^−1^ hypoxanthine, 0.25 mm NaHCO3, 50 µg mL^−1^ gentamicin sulfate and 10% pooled heat‐inactivated O+ human serum. To obtain purified ring and trophozoite stages, an asexual parasite culture was synchronized by repeated sorbitol treatments^[^ ^51^ ^]^ to obtain a synchronization window of approximately 6 h. Ring stage parasites (2.26% parasitemia) were harvested at 10–16 h post RBC invasion and late trophozoites (1.8% parasitemia) at 32–28 h post invasion. To obtain gametocytes, parasite sexual differentiation was induced by overgrowth of an asexual parasite culture, and at the appearance of stage I gametocytes, a 48 h N‐acetyl‐glucosamine (NAG) treatment was used to eliminate residual asexual parasites. After 8 days of cultivation, the gametocyte culture (1.3% parasitemia, stage V of maturation) was centrifuged for 10 min at 3000 rpm on a 30% Percoll cushion in RPMI 1640 to enrich for gametocyte‐infected RBCs. These were subsequently passed through MACS Separation Columns CS (Miltenyi Biotec) to obtain a 95% pure gametocyte preparation. Rings, late trophozoites, and gametocytes, whose morphology was analyzed in Giemsa stained blood smears, were counted and diluted to 50.000 i‐RBCs per µL in whole blood (i.e., a 50% suspension of O+ RBCs, without removal of the buffy coat) to mimic the condition of venous or fingerprick samples. After addition of heparin, parasite samples were diluted tenfold prior to measurements in TMek.

### Sample Preparation and Measurement Procedure on Human Whole Blood Samples

A 8 µL whole blood sample from patients or healthy blood donors, taken with a venous or peripheral sampling, was diluted in 72 µL of a heparin‐PBS solution (20 000 IU L^−1^), to avoid coagulation and achieve a hematocrit around 4% which favored magnetophoretic capture (Figure 3a). This protocol avoids RBCs aggregation, as shown in the optical microscopy image of Figure 3b, which would be highly detrimental to the quantitative assessment of parasitemia. Then, 70 µL of diluted blood was dispensed on a glass slide with a PDMS rectangular gasket (Figure 3c), pre‐charged on a cartridge, defining the bottom and the lateral walls of the magnetophoretic cell. When the operator closed the cartridge lid, the microchip was pressed against the PDM gasket, thus creating the cell sealing and the electrical connections to the chip pads via spring contacts on the cartridge. The cartridge was inserted in the reader (Figure 3d), thus connecting the chip to the front‐end electronics which implemented the high‐frequency impedimetric measurements and transmits digital data to a laptop. Finally, the magnet assembly was placed in close proximity (50 µm) to the rear face of the microchip, using a motorized linear motion, and the measurement protocol described in Figure 2f started. More details on the sample load and test operation can be found in Movie M1, Supporting Information. Note that, in future developments, the chip and the cartridge could be integrated into a miniaturized disposable device.

### Preclinical Study

The study was approved by the Ethics Committee of AO‐Polo Universitario Luigi Sacco, Milan, Italy (Protocol No. 17928/2018/ and 12860/2019) and by the Comité Régional d'Ethique de la Recherche pour la Santé Humaine du Centre (CRERSH/C) of Cameroon (CE N. 1575/CRERSHC/2019). For malaria samples investigated at Sacco Hospital, control tests have been performed by optical microscopy and PCR (Bosphore Genotyping kit v1 Alpha IVD Srl Italy).

During the preclinical study in Cameroon, two TMek systems were installed in the diagnostic laboratory of the Hôpital Saint Luc of Mbalmayo, where all the analyses were carried out in a small room of the diagnostic laboratory, without air conditioning, room temperature up to 40 °C, and humidity around 80%. Most of samples were taken there, but some samples originated also from the Centre Medical Mr. Jean Zoa of Yaoundé and from the Hôpital Central de Mbalmayo. Only patients with suspect of malaria after a first medical consultation were considered in this study. Upon signature of the informed consent and implementation of the anonymity by the local medical personnel, a file for each patient was collected reporting the essential clinical information, as well as the results of the following tests: complete blood count, thick blood smear microscopy with parasitemia quantification by local staff and RDTs (SD BIOLINE MALARIA Ag P.F/PAN, ABBOT) targeting histidine‐rich‐protein II of *P. falciparum* and lactate dehydrogenase (pLDH) of *Plasmodium* species. To this scope, a peripheral (venous) blood sample was taken from each patient, using standard tubes with EDTA anticoagulant, while only in the last days of the campaign a prick‐test version on a limited number of patients was implemented, taking a capillary sample of just 8 µL and immediately diluting it into the solution of PBS and heparin (see Figure 3a). In addition, all slides prepared for microscopy were collected for an independent evaluation of *Plasmodium* species and parasitemia by two microbiologists of the Sacco Hospital, after the experimental campaign. Ten capillary samples were collected at the Hôpital Saint Luc and 108 venous samples from the hospitals listed above. 15 venous blood samples were discarded because of inaccurate sample conservation during transport to the Hôpital Saint Luc or delay time between collection and measurements larger than 16 h, leading to evident hemolysis and/or transformation of hemoglobin in methemoglobin. 18 tests turned out to be invalid after the application of a robust algorithm for data analysis (see Note S7, Supporting Information) so that the 2 × 2 table for the statistical analysis of the results was based on 75 blood samples. The corresponding patients were distributed as follows: 48 females (10 pregnant), 27 males, age‐span from 6 months to 87 years, average age of 25 years with standard deviation of 21 years. Due to the lack of PCR facilities in Cameroon, as reference for the statistical analysis in Table 2, the final classification of “true” malaria cases carried out by Infectious Diseases specialists of Sacco Hospital was used on the basis of: i) three independent evaluations of the parasitemia via microscopy, ii) the complete blood count, iii) the file with the clinical relevant information on the patient filled by Cameroonian doctors (see Note S5 and Dataset D1, Supporting Information). Despite this reference for the statistical analysis is not that usually adopted for RDTs evaluation (PCR), our classification is much more reliable than that carried out using a single microscopy examination. Furthermore, within the large confidence intervals arising from the limited number of samples analyzed in this preliminary study, a major impact on the test features (sensitivity and specificity) of the reference choice with respect to PCR was not expected. Chips were reused many times after cleaning and sterilization by subsequent washing with a sodium dodecyl sulfate solution, water‐ethanol solution, and deionized water.

### Statistical Analysis

The sample composition, methods for the classification of “true malaria cases,” and pre‐processing of data from the prevalidation campaign are described in the previous section and in Note S5, Supporting Information. The evaluation of the test sensitivity and specificity were performed from Dataset D1, Supporting Information, using the MedCalc statistical software. The data analysis algorithm used for TMek classification (positive/negative) is described in Note S7, Supporting Information. Data in Figure 4a,b are from one or two replicates for each concentration and error bars are derived from the signal SD, as explained in Note S7, Supporting Information. The box‐plot of Figure 5 has been produced using MATLAB (MathWorks, USA).

## Conflict of Interest

The authors declare no conflict of interest.

## Supporting information

Supporting Information

Supplemental Movie 1

Supporting Data D1

## Data Availability

Research data are not shared.
