# Supplementary material for: A Lab‐On‐chip Tool for Rapid, Quantitative, and Stage‐selective Diagnosis of Malaria
Source: Adv Sci (Weinh). 2021 May 13;8(14):2004101. doi: 10.1002/advs.202004101 (PMC8292881; doi:10.1002/advs.202004101)
Supplement: Supplementary file 1 — Supporting Information [file ADVS-8-2004101-s003.pdf]

© 2021 Wiley-VCH GmbH

ADVANCED SCIENCE Open Access

Supporting Information

for *Adv. Sci.,* DOI: 10.1002/advs.202004101

A Lab-on-chip Tool for Rapid, Quantitative and Stage-selective Diagnosis of Malaria

*Marco Giacometti, Francesca Milesi, Pietro Lorenzo Coppadoro, Alberto Rizzo, Federico Fagiani, Christian Rinaldi, Matteo Cantoni, Daniela Petti, Edoardo Albisetti, Marco Sampietro, Mariagrazia Ciardo, Giulia Siciliano, Pietro Alano, B. Lemen, J. Bombe, Marie Thérèse Nwaha Toukam, Paul Fernand Tina, Maria Rita Gismondo, Mario Corbellino, Romualdo Grande, Gianfranco Beniamino Fiore, Giorgio Ferrari, Spinello Antinori, Riccardo Bertacco**

Supporting Information

A lab-on-chip tool for rapid, quantitative and stage-selective diagnosis of malaria

*Marco Giacometti, Francesca Milesi, Pietro Lorenzo Coppadoro, Alberto Rizzo, Federico Fagiani, Christian Rinaldi, Matteo Cantoni, Daniela Petti, Edoardo Albisetti, Marco Sampietro, Mariagrazia Ciardo, Giulia Siciliano, Pietro Alano, B. Lemen, J. Bombe, Marie Thérèse Nwaha Toukam, Paul Fernand Tina, Maria Rita Gismondo, Mario Corbellino, Romualdo Grande, Gianfranco Beniamino Fiore, Giorgio Ferrari, Spinello Antinori, Riccardo Bertacco**

Note 1: Implementation of the magnetic field gradient

Estimate of the entity of the magnetic field gradient needed for magnetophoretic separation

Assuming a volume of red blood cells V_{RBC} = 9.1·10^{-11} cm^{-3}, a density of RBC ρ_{RBC} = 1.15 g·cm^{-3} and a plasma density ρ_P = 1.025 g·cm^{-3}, the sum of the weight and Archimede's force on the single RBC, $F_{gb} = (\rho_{RBC} - \rho_P)V_{RBC}g$, is equal to 1.1·10^{-13} N. According to the expression of the magnetic force on a superparamagnetic particle, $F_m = \frac{1}{2}\mu_0 V \Delta\chi \nabla H^2$, assuming a difference in susceptibility $\Delta\chi$ = 1.8·10^{-6} between RBC and plasma,[1] the value of the H^2 gradient needed to balance $F_{gb}$is of the order of 1·10^{15} A^2·m^{-3}.

A similar estimate can be made for a single crystal of hemozoin suspended in plasma. Assuming an average volume V_{HC} = 2.2·10^{-14} cm^{-3}, a density ρ_{HC} = 1.15 g·cm^{-3}, and a difference in susceptibility $\Delta\chi$ = 4.1·10^{-4} compared to plasma, the value of the H^2 gradient needed to balance F_{gb} for hemozoin crystals is of the order of 1.7·10^{13} A^2·m^{-3}.

Characterization of the macroscopic field produced by the permanent magnet assembly

The characterization of the field and gradient produced by the two NdFeB magnets sandwiching a 0.2 mm thick μ-metal foil has been carried out by using a Hall-probe mounted on a linear positioning stage. We measured the field component perpendicular to the surface of the assembly at variable distance from the surface of the magnets, while keeping the probe center in the symmetry plane defined by the μ-metal foil. As a matter of fact, the stray field emanating from the μ-metal is essentially parallel to the foil plane (see Fig. 2b in the main text), i.e. perpendicular to the chip surface, at least within ±1 mm from the symmetry plane, corresponding to the area with measurement electrodes on the chip, centered on the foil plane. At 0.5 mm from the magnet

assembly surface, roughly corresponding to the position of the concentrators taking into account that during measurements the back of the chip (500 μm thick) is placed in close proximity to the magnets, the H field produced is about $6 \cdot 10^5$ A·m^{-1} while the corresponding ∇H^2 is $7 \cdot 10^{14}$ A^2·m^{-3}. These values are definitely enough to saturate the Ni concentrators (see loops below) and produce the macroscopic attracting force towards the chip acting on i-RBC, responsible for the deviation from the vertical of their trajectories (see Fig. 4e in the main text).

The effect of the μ-metal foil has been evaluated by measuring the field and gradients produced with and without the foil, while keeping the same distance of 0.2 mm between the faces of the magnets. It turns out that the field is very similar (within 5%), while the mechanical stability of the assembly largely improves with the foil because we can better press the magnets using the foil as spacer and avoid the sliding motion due to the magnetic repulsive force.

M(H) behavior of fabricated Ni concentrators

Figure S1 reports the characteristic M(H) curve of the out-of-plane component of the magnetization vs. the out-of-plane magnetic field, measured on a portion of the microchip with 1000 fabricated Ni concentrators arranged in a hexagonal lattice, using a Vibrating Sample magnetometer. The out-of-plane direction has been chosen because the field created by the permanent magnet assembly is applied mainly perpendicularly to the chip surface.

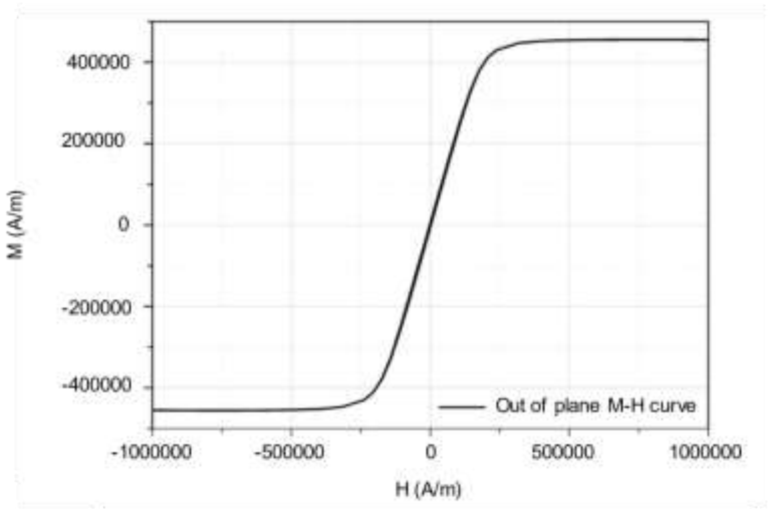

Figure S1: **Magnetic characterization of Ni concentrators.** Measured M(H) curve for the microfabricated Ni concentrators

The experimental curve shows that, in order to saturate the magnetization (M), an external field of about 300000 A·m^{-1} is required. As discussed above, the field provided by the permanent magnet

assembly is 600000 A·m^{-1}, thus ensuring that all Ni posts are saturated upon the magnet approach during the assay. Furthermore, the M(H) curve displays a negligible hysteresis and remanent magnetization. This is crucial for TMek operation, because the measurement protocol (see Fig. 2f of the main text) exploits the i-RBC release upon permanent magnet disengagement, which is based on the absence of sizable ferromagnetic remanence. The saturation magnetization is about 4.5·10^{5} A·m^{-1}, in nice agreement with the theoretical value of 5·10^{5} A·m^{-1}, taking into account some uncertainty in the determination of the Ni post height due to some non-uniformity in the microfabrication process.

Concentrators layout

Ni concentrators are arranged in a hexagonal lattice, as depicted in Figure S2. The lattice parameter (S) is 160 μm, while the diameter and height of each cylinder are 40 μm and 20 μm, respectively.

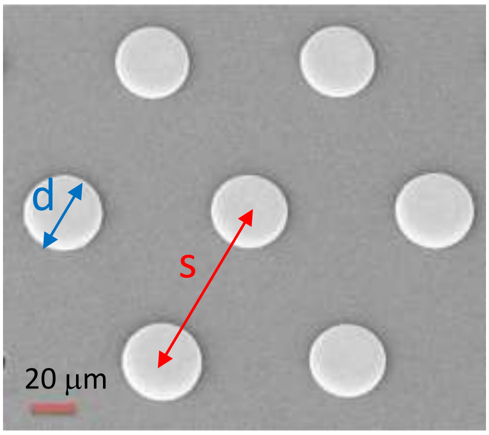

Figure S2: Layout of Ni concentrators. Secondary Electron Microscopy image of microfabricated Ni concentrators. The lattice parameter (S) is 160 μm, while the diameter (D) is 40 μm.

Note 2: Chip fabrication

The process flow is described in more details in Figure S3.

Optimized electroplating conditions corresponds to a working temperature of 50 degree and a pH level of 4.0 as recommended by the ATOTECH. The current density ensuring good hole filling is 1.4 A·dm^{-2}.

Mechanical polishing is carried out between steps 5 and 6, in order to achieve a roughness lower than 50 nm rms.

A uniform gold coating is finally deposited on the back on the chip to create a reference ground plane and reduce the background.

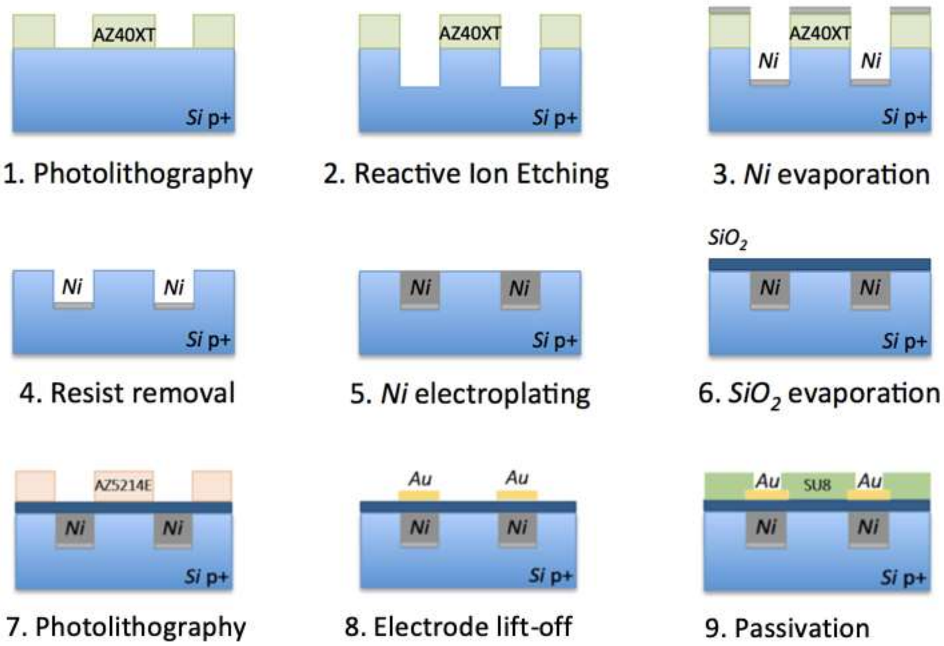

Figure S3: **Chip fabrication.** Process for the fabrication of TMek chips

Note 3: Impedimetric detection and multiphysical simulations

According to the Maxwell mixture theory[2] the conductivity of the medium is modified by the presence of insulating particles according to the following expression:

$$\sigma_{mix} = \sigma_m(1 - \frac{3}{2}\phi) \qquad (1)$$

where ϕ represents the volume fraction of the particles in the medium. For small values of the volumetric fraction the equation above can be rewritten in terms of resistivity:

$$\rho_{mix} = \rho_m(1 + \frac{3}{2}\phi) \qquad (2)$$

The total resistance percentage variation due to the capture of a number N_p of particles, each of them with volume V_p, within the electrode probing volume, is thus given by:

$$\frac{\Delta R}{R_0} = \frac{3}{2}\frac{N_p V_p}{V_e} \qquad (3)$$

where V_e is the volume sensed by the electrodes, approximately given by area of the electrodes multiplied by their separation.

Each sensor, as described in the main text (Methods - Electronic platform for impedimetric detection and data acquisition), is composed by two areas: a measurement area, in which the magnetic concentrators are located, and a reference area. The inner elements of the electrodes from both areas are connected together (Fig. S4) to the virtual ground of a transimpedance amplifier

(TIA) in order to implement an analog differential measurement enabling both the partial compensation of the common fluctuations and the removal of the offset.

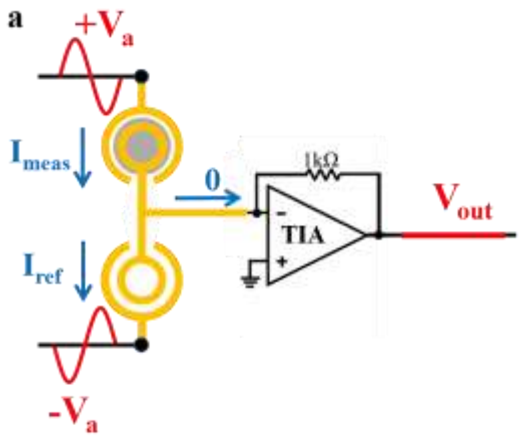

Figure S4: **Working principle of the differential measurement.** The measurement electrodes (top) and the reference electrodes (bottom) are stimulated with counter-phase signals. Without i-RBCs captured by the magnetic concentrators (a) the measured current is ideally zero. The capture of i-RBC (b) produces a net differential signal.

When two counter-phase sinusoidal signals with amplitude V_a= 100 mV are applied to the outer elements of the electrodes in the measurement and reference areas, the TIA measures the resulting differential current proportional to the impedance mismatch between the measurement and reference electrodes, originated by the capture of i-RBCs.

The differential current variation is thus given by:

$$\Delta I = -V_a \frac{\Delta R}{R_0^2} \qquad (4)$$

where R_0 = 170 Ω is the initial resistance of the electrodes and ΔR the impedance mismatch due RBC capture. This is the expression which has been used to evaluate the impedance variation from the COMSOL simulation of the t-RBC capture and detaching, by monitoring over time the number of particles N_p which contribute to the impedance variation according to equation (3).

In figure S5 we report the simulated current variations for the three values of parasitemia considered in the text: 0.01%, 0.1% and 1%, from which the signal amplitudes corresponding to black squares in Figure 4a of the main text have been obtained.

The discretized behavior of the signals is due to the limited portion of the chip used in the numerical simulations.

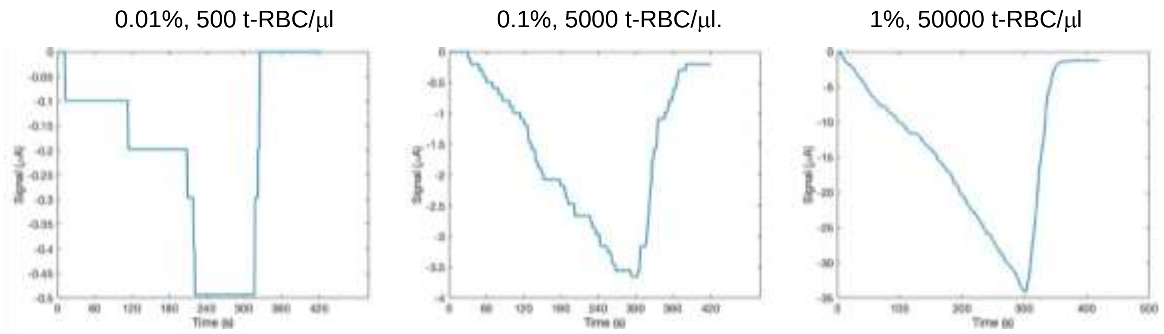

Figure S5: **COMSOL simulation of experiments on t-RBCs.** Simulated current signals during the first capture and release process for different values of parasitemia (in percentage) and t-RBC concentration.

Note 4: Sensitivity to free hemozoin crystals

We used synthetic hemozoin crystals (β-hematin) from Invivogen suspended in PBS at calibrated concentrations to estimate the test sensitivity to free hemozoin crystals, in analogy to what has been done for t-RBCs. In Figure S6 we show a typical differential signal taken using a protocol similar to that used for i-RBCs and described in the main text, after subtraction of a linear drift. Upon signal stabilization, at 60 s we approach the magnet and the current decreases due to HC accumulation. When we re-engage the magnet, at 200 s, HC are released and the initial base-line is recovered. The HC concentration here was 15 µg/ml, and the corresponding signal is about 300 nm. By decreasing the concentration, the lowest detectable limit is mainly determined by the tendency of hemozoin to stick on the concentrators and the difficulty to evaluate net signals over the signal drift. We found distinguishable signals, on the order of 50 nA, down to 3 µg/ml, which corresponds to the amount of hemozoin contained in about 5000 i-RBC/µl.

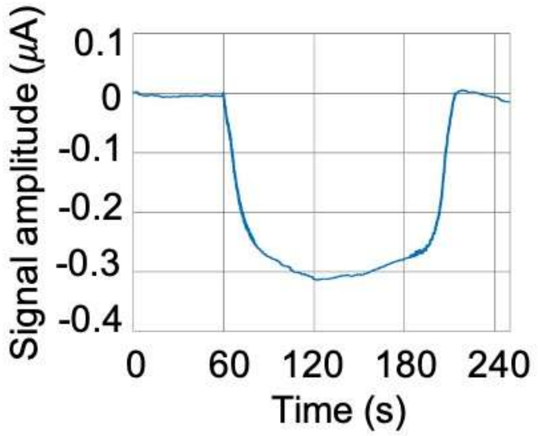

Figure S6: Current signal measured on a suspension of β-hematin crystals with concentration of 15 µg/ml, upon subtraction of a linear drift.

Note 5: Control tests and classification

86 venous blood samples from patients enrolled in the pre-validation campaign in Cameroon were evaluated for diagnosis of malaria also in Italy. Every patient was tested for malaria parasites by microscopic examination of thick smears slides prepared in Cameroon and stained with May Grunwald Giemsa. Automated cell counting was performed on venous samples at the Hopital Saint Luc of Mbalmayo, in order to normalize the i-RBC counts and have a general clinical assessment of the blood sample. All the slides collected were evaluated by two expert microscopists from the CLIMVIB (CLInical Microbiology, VIrology and Bioemergency) Laboratory Unit of Sacco University Hospital in Milan (Italy). Bench aids for the diagnosis of malaria infections (WHO)[3], Atlas of Human Parasitology[4] and Atlas of Human Malaria[5] were used as references for the identification of malaria parasites. The results of the microscopy tests carried out at Sacco Hospital, together with those obtained in Caemeroon, results were provided to Infectious Diseases specialists of Sacco Hospital for the final classification of “true” malaria cases. Thick smears were examined by optical microscopy using 100x oil immersion objective (total magnification 1000x: 100x objective multiplied by 10x of the eyepiece). To evaluate the levels of parasitemia of samples collected in Cameroon we used thick smears formula[6], where WBCs stands for white blood cells:

No. Parasites x (No. of WBCs/µL ÷ No.WBCs counted) = No. parasites/µL of blood

The data about WBC/µl were collected by automated cell counters in Cameroon. All the parasitologists evaluated 100 microscopic fields for slide.

Three other patients were enrolled in Italy by the inpatients of the Sacco University Hospital. They were diagnosed with malaria by microscopy: blood thin film and thick smear stained with May Grunwald Giemsa (MGG Quick Stain – Bio-Optica ITA). Results were confirmed by loop-mediated isothermal amplification (LAMP) (Illumigene, Meridian Bioscience™ ITA) and real-time PCR (Alfa diagnostici ITA).

To determine the percent of infected RBCs in patients from Sacco University Hospital we used the thin smear method.

Note 6: On-field quantification capabilities

Figure S7 displays the amplitude signal A_1 versus the parasitemia of infected human blood samples resulting from the prevalidation campaign carried out in Cameroon. Here we consider only positive venous samples taken at the Hôpital Saint Luc of Mbalmayo. We discarded samples arising from other hospitals, due to the low control on the sample conservation during transport which can induce spurious hemoglobin transformations detrimental to the present analysis. The error bar on

the parasitemia reported in Fig. S7 is given by the difference of the extreme values estimated by three microscopists operating in double bind mode.

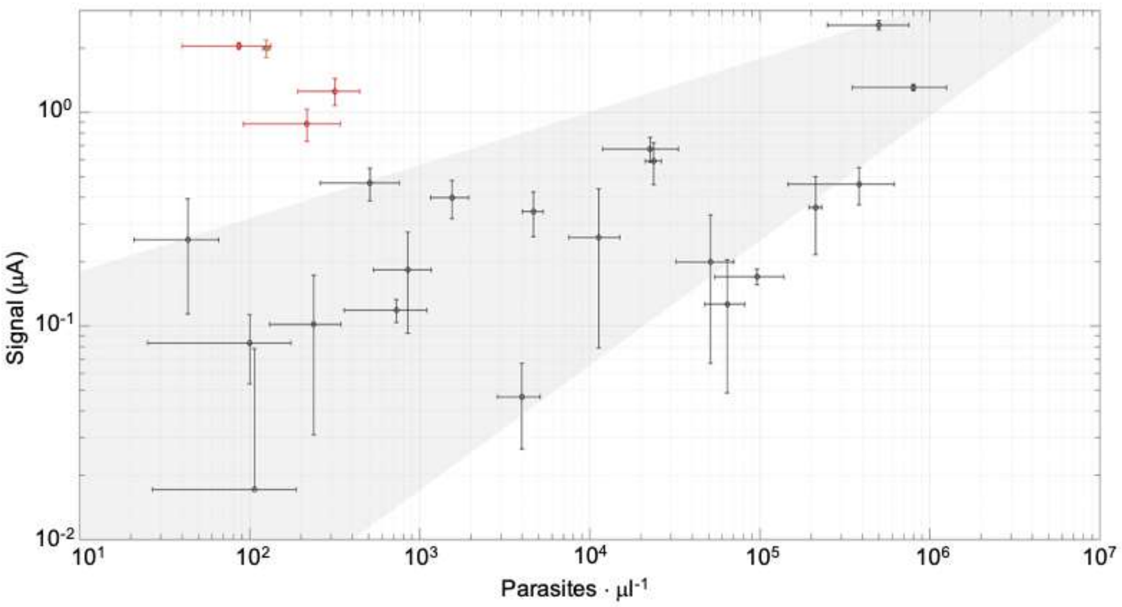

Figure S7: On-field quantification. TMek signal amplitude (A_1) vs. parasitemia estimated via microscopy. The vertical error bars are evaluated as explained in the methods of main text. Horizontal error bars are the difference of the extreme values estimated by three microscopists. Black dots display a moderate linear correlation with parasitemia, graphically indicated by the shaded grey area containing all experimental points when the error bars are considered. Red dots correspond to samples containing a sizable concentration of gametocytes and free pigment.

A moderate linear correlation is seen for black dots, with a Pearson correlation coefficient of 0.51, mainly due to the large error bars both on the parasitemia and the signal amplitude, the latter also associated to the use of different chips and setups in the on-field tests. Because of some irreproducibility in the fabrication of the first lot of chips, as well in the setting of the experimental conditions using different setups, the sensitivity to a given concentration of i-RBCs was largely dependent on the combination chip-setup. Despite a normalization factor of the experimental amplitudes (based on the comparison of signals taken on reference samples made of t-RBCs) has been introduced, the signal amplitude A_1 reported in Fig. S7 is thus affected by an additional error bar which cannot be easily estimated. This is at variance with the calibration curves reported in Fig. 4 of the main text, referring to experiments carried out with the same chip and setup. However, laboratory results on t-RBCs indicate that the on-field quantification capabilities on real patients can be largely improved upon careful engineering of the measurement apparatus and chip-production scale-up, moving from first prototypes to lots of identical devices.

Note also that a second population of results (empty red dots in Fig. S7) deviates from linearity, but these anomalous data correspond to samples where microscopy analysis revealed the presence of either a lot of free pigment or gametocytes. The presence of these corpuscles with higher magnetic susceptibility and capture efficiency with respect to i-RBCs in the asexual stage (see Table 1), gives rise to an anomalous increase of the TMek signal, in particular for gametocytes which have a larger volume with respect to HC. However, as shown in Figure 5f of the main text, the presence of a sizable amount of gametocytes can be detected looking at the signal waveform during capture, without microscopy investigation. Similar considerations apply also to free-pigment,[7] thus enabling in perspective a self-consistent use of TMek.

Note 7: **Data analysis algorithm used for the prevalidation campaign**

In order to ensure a robust data analysis compatible with automatic classification we developed an algorithm based on three steps: (1) determination of invalid tests, (2) evaluation of the signal amplitude and (3) test classification (positive/negative).

1. A test is considered invalid if at least one of the following conditions applies to the recorded signal, considering only the first peak (A1) : (i) the background due to signal drift evaluated before the magnet disengagement (between 250 and 300 s) and just before the re-engagement (between 550 and 600 s) either display a change of sign of its slope or a variation of the magnitude of its slope larger than a factor five; (ii) in case of small signals (with amplitude smaller than three times the blank signal measured that day on a reference PBS sample), presence of spurious fluctuations between 300 and 600 s or peaks in correspondence to the magnet motion opposite to that expected and with amplitude larger than the blank; (iii) in case of small signals, background with upward concavity; (iv) fluctuations or steps within the first peak (between 300 and 600 s) with amplitude larger than 2 μA;

2. The evaluation of the amplitude A_1 is carried out by considering a linear background between 300 s and 600 s and measuring the signal at 420 s with respect to the background, as shown in Fig. 2f. The net signal ΔA is then obtained by subtracting the amplitude A_{1w} of the blank signal of the day, measured with the same chip and setup on a reference PBS sample: $\Delta A = (A_1 - A_{1w})$. The associated error is estimated starting from the evaluation of the error σ_1 of A_1 (square root of the sum of the squares of the signal standard deviations in the 250-300 s and 370-420 s intervals) and the error σ_w of the blank signal. For the latter we assume that the error on the blank amplitude is on the same order of the blank amplitude itself, due to some irreproducibility in the blank measurement which depends on spurious coupling induced by the magnet motion: $\sigma_w = A_{1w}$. The total error on the net signal ΔA is thus $\sigma = (\sigma_1^2 + \sigma_w^2)^{1/2}$.

3. Finally, a test is classified as positive if $\Delta A > \sigma$.

Note 8: Data analysis for laboratory tests on t-RBCs

For tests performed on blood samples with treated RBCs mimicking i-RBCs, we used a data analysis protocol very similar to that presented above. The amplitude A_1 is evaluated by considering a linear background between 300 s and 600 s and measuring the signal at 420 s with respect to the background, as shown in Fig. 2f. The net signal ΔA is then obtained by subtracting the amplitude A_{1HB} of the blank signal of the day, measured with the same chip and setup on a reference sample just made of the healthy donor whole blood diluted in PBS and heparin: $\Delta A = (A_1 - A_{1HB})$. Due to high repeatability of blank measurements in good laboratory conditions, in this case the error (σ) associated to ΔA is just given by the square root of the sum of the squares of the errors evaluated on A_1 and A_{1HB} as explained above. The lowest detectable concentration is estimated by assuming that a meaningful signal must fulfil the condition $\Delta A > \sigma$.

Additional information

Movie M1 (separate file). A movie showing in detail the sample load and test operation is available online.

Dataset D1 (separate file). Dataset with anonymous patient IDs, clinical data, three independent evaluation of the parasitemia by independent microscopists, the malaria classification carried out by an infectious disease specialist on the basis of the above data, the results of a RDT (SD Bioline) and TMek carried out in parallel**.**

REFERENCES

[1] K. Han and A. B. Frazier, J. Appl. Phys. 96 (10), 5797 (2004)

[2] Sun, Tao, and Hywel Morgan. Single-Cell Microfluidic Impedance Cytometry: A Review. Microfluidics and Nanofluidics 8, n. 4 (aprile 2010): 423-43.

[3] World Health Organization. (2000). Bench aids for the diagnosis of malaria infections, 2nd ed. World Health Organization. https://apps.who.int/iris/handle/10665/42195 (date of access: 08 october 2020)

[4] John Williams, Atlas of Human Parasitology, fifth edition Lawrence R. Ash, Thomas C. Orihel. American Society for Clinical Pathology Press, Chicago, IL, 2007. xvi + 540 pp., hardback, ISBN: 0-89189-1676

[5] Giovanni Swierczynski, Maria Gobbo, Atlas of Human Malaria (Atlante della Malaria Umana), *Journal of Travel Medicine*, Volume 15, Issue 2, 1 March 2008, Pages 143–144, https://doi.org/10.1111/j.1708-8305.2008.00196.x

[6] Laboratory diagnosis of malaria. Determination of Parasitemia. DPDx, CDC, https://www.cdc.gov/dpdx/resources/pdf/benchAids/malaria/Parasitemia_and_LifeCycle.pdf (date of access: 08 october 2020)

[7] F. Milesi et al. , On-Chip Selective Capture and Detection of Magnetic Fingerprints of Malaria, Sensors, 20, 4972 (2020)
